# Combined Oxygen-Enhanced MRI and Perfusion Imaging Detect Hypoxia Modification from Banoxantrone and Atovaquone and Track Their Differential Mechanisms of Action

**DOI:** 10.1158/2767-9764.CRC-24-0315

**Published:** 2024-10-01

**Authors:** James P.B. O’Connor, Victoria Tessyman, Ross A. Little, Muhammad Babur, Duncan Forster, Ayşe Latif, Susan Cheung, Grazyna Lipowska-Bhalla, Geoff S. Higgins, Marie-Claude Asselin, Geoff J.M. Parker, Kaye J. Williams

**Affiliations:** 1 Division of Radiotherapy and Imaging, The Institute of Cancer Research, London, United Kingdom.; 2 Division of Cancer Sciences, University of Manchester, Manchester, United Kingdom.; 3 Department of Radiology, The Christie NHS Foundation Trust, Manchester, United Kingdom.; 4 Division of Pharmacy and Optometry, University of Manchester, Manchester, United Kingdom.; 5 Cancer Research UK Manchester Centre, University of Manchester, Manchester, United Kingdom.; 6 CRUK/MRC Oxford Institute for Radiation Oncology and Biology, University of Oxford, Oxford, United Kingdom.; 7 Division of Informatics, Imaging and Data Sciences, University of Manchester, Manchester, United Kingdom.; 8 Bioxydyn Ltd., Manchester, United Kingdom.; 9 Centre for Medical Image Computing, University College London, London, United Kingdom.

## Abstract

Oxygen-enhanced MRI (OE-MRI) has shown promise for quantifying and spatially mapping tumor hypoxia, either alone or in combination with perfusion imaging. Previous studies have validated the technique in mouse models and in patients with cancer. Here, we report the first evidence that OE-MRI can track change in tumor oxygenation induced by two drugs designed to modify hypoxia. Mechanism of action of banoxantrone and atovaquone were confirmed using *in vitro* experiments. Next, *in vivo* OE-MRI studies were performed in Calu6 and U87 xenograft tumor models, alongside fluorine-18–fluoroazomycin arabinoside PET and immunohistochemistry assays of hypoxia. Neither drug altered tumor size. Banoxantrone reduced OE-MRI hypoxic fraction in Calu6 tumors by 52.5% ± 12.0% (*P* = 0.008) and in U87 tumors by 29.0% ± 15.8% (*P* = 0.004) after 3 days treatment. Atovaquone reduced OE-MRI hypoxic fraction in Calu6 tumors by 53.4% ± 15.3% (*P* = 0.002) after 7 days therapy. PET and immunohistochemistry provided independent validation of the MRI findings. Finally, combined OE-MRI and perfusion imaging showed that hypoxic tissue was converted into necrotic tissue when treated by the hypoxia-activated cytotoxic prodrug banoxantrone, whereas hypoxic tissue became normoxic when treated by atovaquone, an inhibitor of mitochondrial complex III of the electron transport chain. OE-MRI detected and quantified hypoxia reduction induced by two hypoxia-modifying therapies and could distinguish between their differential mechanisms of action. These data support clinical translation of OE-MRI biomarkers in clinical trials of hypoxia-modifying agents to identify patients demonstrating biological response and to optimize treatment timing and scheduling.

**Significance:**

For the first time, we show that hypoxic fraction measured by oxygen-enhanced MRI (OE-MRI) detected changes in tumor oxygenation induced by two drugs designed specifically to modify hypoxia. Furthermore, when combined with perfusion imaging, OE-MRI hypoxic volume distinguished the two drug mechanisms of action. This imaging technology has potential to facilitate drug development, enrich clinical trial design, and accelerate clinical translation of novel therapeutics into clinical use.

## Introduction

Tumor hypoxia is an adverse prognostic factor in patients with solid tumors. It also impacts negatively on response to radiotherapy and chemotherapy (1). Both high pretreatment levels of hypoxia (2, 3) and subsequent persistence of hypoxia during therapy (4) predict poor outcome. More recently, the negative impact of hypoxia on response to targeted therapies and immunotherapies has been recognized (5, 6). Consequently, there is interest in developing therapeutic approaches that modify hypoxia. Strategies include improving oxygen delivery through inducing vasodilation (7), reducing oxygen demand within tumor tissues (8), and selectively targeting hypoxia using prodrugs that become cytotoxic when activated in hypoxic tumor subregions. Evidence that these strategies are beneficial includes a randomized phase III placebo-controlled trial reporting how addition of the hypoxic radiosensitizer nimorazole to radical radiotherapy improved loco-regional control of supraglottic and pharyngeal cancer (9).

Optimal development of hypoxia-modifying therapies requires biomarkers that identify which tumors are hypoxic prior to starting standard-of-care therapy and can also detect successful hypoxia modification on/after therapy (10). Unfortunately, few hypoxia biomarkers are validated, available, and affordable. This has hindered rational drug development and clinical trial evaluation of hypoxia-modifying therapies (11). Gene and protein expression signatures show promise in stratifying patients but are not yet validated for routine clinical use (12). Further, these signatures and histopathology biomarkers (13) sub-sample tumors and are limited to single measurements obtained from initial diagnostic biopsy (14).

Imaging technologies have multiple advantages over the above techniques. Imaging enables serial, non-invasive, whole tumor sampling of multiple lesions within a given patient (15). For example, positron emission tomography (PET) studies using fluorine-18 [^18^F]-based tracers have provided proof-of-concept that imaging can track biological effects of hypoxia-modifying therapeutics in preclinical models (16). Further, PET studies have demonstrated hypoxia-modifying effects of chemo-radiotherapy in patients (4). However, despite these encouraging data (11) the cost, limited availability and lack of standardization of hypoxia PET imaging has precluded widespread use to select patients in phase III evaluation of hypoxia-modifying therapeutics (17).

A potential alternative imaging technology is oxygen-enhanced MRI (OE-MRI). This technology measures the change in longitudinal relaxation rate (*R*_1_) of tissue protons induced by switching between breathing air and 100% oxygen (or a similar gas, carbogen composed of 95%–98% O_2_ and 2%–5% CO_2_) during MRI scanning (18). Recent work suggests that this technique—when combined with gadolinium-based dynamic contrast-enhanced MRI (DCE-MRI)—can quantify and spatially map tumor hypoxia *in vivo*, making it a viable alternative to PET imaging (19). OE-MRI has been validated with immunohistochemistry assays of hypoxia in multiple xenograft models (20, 21) and in human tumors (22). Further, OE-MRI can map and track hypoxia modification induced by single-agent radiotherapy and combined chemo-radiotherapy in xenograft models of cancer and in patients with non–small cell lung cancer (NSCLC; ref. 23).

Here, we report the first evidence that non-invasive OE-MRI can identify, quantify, and map the effects of drugs designed to modify hypoxia. Using two xenograft models, we evaluated banoxantrone (AQ4N) that is reduced under hypoxic conditions to AQ4, a potent topoisomerase II inhibitor causing direct killing of hypoxic cells (24) and atovaquone that decreases the oxygen consumption rate (OCR) in tumors by inhibiting mitochondrial complex III of the electron transport chain (25, 26). MRI data were compared to [^18^F]-fluoroazomycin arabinoside ([^18^F] FAZA) PET data and validated using immunohistochemistry readouts of hypoxia. We show that mapping tumor regional heterogeneity (27) with combined OE-MRI and DCE-MRI provides insight into the differential drug mechanisms of action, beyond that available from [^18^F] FAZA PET. Collectively, these data provide a rationale for clinical use of OE-MRI, combined with perfusion imaging, to accelerate clinical translation of hypoxia-modifying therapies in future clinical trials.

## Materials and Methods

### *In vitro* experiments

Banoxantrone (dihydrochloride salt, Abcam) and atovaquone (Tokyo Chemical Industry UK) stocks were prepared at 10 and 20 mmol/L, respectively, in DMSO and stored at −20°C.

Human NSCLC (Calu6; RRID:CVCL_C8GA) and glioblastoma (U87MG: RRID:CVCL_0022) cells (purchased from ATCC with authenticity confirmed by short tandem repeat profiling) were cultured in RPMI 1640 medium, supplemented with 10% fetal bovine serum and 2 mmol/L L-glutamine, and maintained in standard culture conditions (95% air/5% CO_2_ at 37°C) in a humidified incubator. HCT116 (RRID:CVCL_0291), KHT, MDA-MB-231 (RRID:CVCL_0062) cells were used in a screening step, with xenografts generated using equivalent culture. All cell lines were tested for mycoplasma regularly.

#### Drug exposure

Exponential phase Calu6 cells were plated at 3 × 10^5^ cells/well onto sterile coverslips (AQ4 fluorescence) or directly into six-well plates (clonogenics). Following overnight culture in standard conditions, cells were exposed to normoxia (21% O_2_) or hypoxia (1% O_2_ or 0.1% O_2_; Whitley H35 Hypoxystation, Don Whitley Scientific Ltd.) for 3 hours before treatment with drug/vehicle control for a further 24 hours.

#### AQ4 fluorescence

Cell monolayers were cultured on coverslips, washed with PBS, and fixed in 10% formalin for 20 minutes. Following three PBS washes, cell nuclei were counterstained with 4′,6-diamidino-2-phenylindole (DAPI; 1:2,500 in PBS for 1-minute exposure) followed by a further PBS wash. Coverslips were then mounted onto slides using DAKO fluorescent mounting media (DAKO-Agilent). Images were acquired on a 3D-Histech Panoramic-250 microscope slide-scanner using a 20×/0.30 Plan Achromat objective system (Zeiss UK) with DAPI and (tetramethylrhodamine) TRITC (for AQ4) filter sets. Snapshots of the slide-scans were taken using the Case Viewer software (3D-Histech). Fluorescent signal intensity was analyzed from ∼100 nuclei per sample from three random fields using ImageJ (NIH: RRID:SCR_003070).

#### Clonogenic survival assays

Following exposure to drug or vehicle, cells were harvested, counted, and re-seeded into six-well plates at various seeding densities (100–3,200 cells/well), then incubated for 12 days under standard culture conditions (95% air/5% CO_2_ at 37°C) in standard medium (no additional drug/vehicle; no change of culture medium during the 12-day incubation period) until colonies of >50 cells were present. Colonies were fixed and stained with 0.5% methylene blue in 70% methanol and counted manually. Colony formation efficiencies were normalized to the relevant DMSO control to give surviving fractions.

#### Metabolic phenotype

Mitochondrial specific OCR (pmoles O_2_/minute) was measured using a Seahorse XF^e^96 Analyzer (Agilent Technologies) according to manufacturer’s protocol in normoxia (21% O_2_) or hypoxia (3% O_2_; Whitley H35 Hypoxystation with Whitley i2 Instrument Workstation Don Whitley Scientific Ltd.). Assay optimization was performed as described previously to provide reproducible changes in OCR and restoration to the ambient oxygen concentrations between measurements [125–105 and 20–12 mm Hg normoxia/hypoxia, respectively (28)]. Calu6 cells were seeded at 12,000 cells for normoxic and 8,000 cells for hypoxic conditions into 96-well Seahorse microplates. OCR measurements were performed after 24 hours drug/vehicle treatment in normoxia or hypoxia. Mitochondrial specific OCR was determined by subtracting OCR measured following rotenone and antimycin A injection from the basal OCR measurement. OCR results were normalized to cellular protein density determined by sulforhodamine B (SRB) assay.

### *In vivo* experiments

All *in vivo* studies complied with UK guidelines on animal welfare in cancer research (29) and had institutional board and regulatory ethical approval under Home Office license PPL707760 (granted to K.J. Williams). Tumors were propagated by injecting 0.1 mL of Calu6 cells (2 × 10^7^ cells/mL in Matrigel) or 0.1 mL of U87 cells (5 × 10^6^ cells/mL in matrigel) subcutaneously on the lower back of female CD-1 nude mice (RRID:IMSR_CRL:022), age 10 to 14 weeks and weighing 20 to 24 *g*. Mice were maintained under a dawn-till-dusk 12 hours light/dark cycles at an average ambient temperature of 21°C (range 19°C–23°C), relative humidity average 55% (range 45%–65%) in pathogen-free housing (Techniplast GM500 Mouse IVC Green Line) with sterilized bedding, environmental enrichment, and access to food water *ad libitum* with water supplied by hydrocpac watering system. Details of the number of mice used for each experiment are in Supplementary Fig. S1A–S1D. Weights, body condition, and behavior were monitored routinely throughout the experiments.

#### Drug exposure

For the banoxantrone experiments, mice received an intraperitoneal injection of either saline (vehicle control) or 60 mg/kg AQ4N in saline (drug treated; ref. 30). For the atovaquone experiment, mice received 2% DMSO and 0.1% carboxymethyl-cellulose in drinking water, either alone (vehicle control) or supplemented with atovaquone (0.2 mg/mL; equivalent to 50 mg/kg/day dosing based on 20 g mouse consuming 5 mL/day; drug treated). Preclinical studies have shown effects of banoxantrone *in vivo* within 3 days (30, 31). In distinction, atovaquone-mediated effects on OCR in tumors are observed within 7 days (25). Therefore *in vivo* experiments were designed to detect changes in xenograft tumor hypoxia within these timeframes.

#### PET data acquisition and analysis

Mice bearing tumors of >200 mm^3^ by caliper measurement were anaesthetized using 1% to 2% isoflurane carried in 100% oxygen. PET scans were performed using an Inveon preclinical PET/CT scanner (Siemens). Mice were injected with approximately 25 MBq of [^18^F] FAZA (synthesized in-house) via a tail-vein catheter under anesthesia. After waking and breathing room air, mice were anesthetized 4 hours later and placed in the Minerve Small Animal Environment System animal bed (Bioscan Inc.) and list mode data were collected for 20 minutes starting at 240 minutes post injection. Anesthesia was maintained during image acquisition via a nose cone with respiration and temperature monitored throughout. After first imaging, animals were recovered in a warmed chamber and allocated into vehicle and treatment groups to give matched tumor volumes. Mice underwent PET [^18^F] FAZA scanning again under the same protocol 1 and 3 days after banoxantrone treatment and 7 days after atovaquone treatment.

Images were reconstructed as a single 20-minute frame using the 3D-OSEM/ MAP algorithm (four OSEM3D iterations and no MAP iterations, with resolution of 1.5 mm; ref. 32). An experienced research PET scientist (DF, 11 years’ experience) drew volumes of interest (VOI) manually over each tumor on the CT image, along with a section of leg muscle bilaterally to normalize the tumor radiotracer uptake to a non-hypoxic region and derive the tumor-to-muscle ratio (TMR). Analysis took place using the Inveon Research Workplace software (Siemens).

#### MRI data acquisition and analysis

For MRI acquisition, when tumors reached >200 mm^3^ by caliper measurement, a different cohort of mice were anesthetized using 1% to 2% isoflurane carried in medical air (21% oxygen) through a nose cone, delivered at 15 L/minutes. Core temperature was controlled at 36°C. Imaging was performed on a 7T Magnex instrument (Magnex Scientific Ltd.) interfaced to a Bruker Avance III console and gradient system (Bruker Corporation) using a surface coil. Localizer and coronal *T*_2_-weighted images were acquired to determine anatomy.

OE-MRI was performed using a variable flip angle (VFA) spoiled gradient echo (SPGR) acquisition to calculate native tissue *T*_1_ (TR/TE = 30/1.44 ms; α = 5°/10°/30°, five averages). Next, 42 dynamic *T*_1_-weighted SPGR acquisitions were acquired (α = 30°; 28.8 seconds temporal resolution) with 18 acquisitions on medical air followed by 24 acquisitions on 100% oxygen, delivered at 15 L/minutes.

DCE-MRI was performed using a VFA SPGR acquisition to calculate native tissue *T*_1_ (TR/TE = 6.02/1.44 ms; α = 2°/5°/10°, 5 averages), followed by 96 dynamic *T*_1_-weighted SPGR acquisitions (α = 10°; 5.8 seconds temporal resolution) with Gd-DOTA injected into a tail vein after 24 acquisitions.

Both OE-MRI and DCE-MRI were acquired in the coronal plane and were matched spatially with a 64 × 64 matrix, 32 × 32 mm field of view and 16 slices, each 1 mm thick. After first imaging, animals were recovered in a warmed chamber and allocated into vehicle and treatment groups to give matched tumor volumes.

A consultant radiologist (JOC, 15 years’ experience MRI research) drew the VOIs. Oxygen-enhancing (*Oxy-E*) voxels were defined by significant increase in *R*_1_ in the last 12 of the 24 dynamic time points acquired on 100% oxygen breathing compared with the *R*_1_ in the 18 baseline time points. The dynamic *R*_1_ measurements were calculated from dynamic SPRG signal intensity changes and the native *T*_1_ measurement, following standard methods. Significance was determined using a *t* test for each voxel time series with significance assumed at *P* < 0.05 and uncorrected for multiple comparisons. Voxels without significant oxygen enhancement were termed oxygen refractory (*Oxy-R*). Mice breathing air (no oxygen challenge; Supplementary Fig. S2A) were used to demonstrate that tumors without oxygen enhancement could be readily distinguished from tumors exhibiting significant oxygen enhancement following gas challenge (Supplementary Fig. S2B).

Previous studies validated *Oxy-R* volume as a biomarker of tumor hypoxia but highlighted that this biomarker is most closely related to tissue hypoxia when measurements were restricted to perfused tumor (20, 21, 23). Therefore, we used DCE-MRI combined with OE-MRI to identify three types of tumor tissue: (i) tumor voxels that were non-perfused (where the DCE-MRI IAUC_90_ ≤0 mmol.s, irrespective of OE-MRI signal; hereafter termed “MRI necrotic” tumor); (ii) perfused tumor voxels (where IAUC_90_ >0 mmol.s) that were refractory to oxygen enhancement (*perfused Oxy-R*; hereafter termed “MRI hypoxic” tumor); and (iii) perfused tumor voxels (where IAUC_90_ >0 mmol.s) that showed oxygen enhancement (*perfused Oxy-E*; hereafter termed “MRI normoxic” tumor). This analysis approach is summarized in Supplementary Fig. S3. All MRI data were examined using established quality control and assurance processes to confirm that contrast agents (oxygen or Gd-DOTA) had been administered successfully in both OE-MRI and DCE-MRI sequences.

For initial experiments comparing MRI with PET and pathology, we calculated MRI hypoxic fraction (ratio of perfused tumor voxels that were refractory to oxygen enhancement to the total number of tumor voxels) as a biomarker of hypoxia as this was more comparable to the pathology gold standard of pimonidazole adduct formation hypoxic fraction. For later experiments tracking changes in tumor subregion habitats, we calculated MRI hypoxic, normoxic, and necrotic volumes.

### *Ex vivo* analysis

Pathology analysis was performed in mice undergoing MRI on their terminal scan (Supplementary Fig. S1A–S1D). Mice received an intraperitoneal injection of 60 mg/kg pimonidazole (hypoxyprobe, Hypoxyprobe Inc.) approximately 55 minutes before 100% oxygen inhalation began for OE-MRI to enable maximal bioreduction of pimonidazole in hypoxic tumor regions. Next, tumors were excised whole and bisected along the imaging plane to match the cut surface to the MRI slice orientation. Specimens were then fixed in 4% neutral buffered formalin for 24 hours, transferred to 70% ethanol, processed, and then embedded in paraffin. Tissue sections 4-µm thick were cut, floated out on a water bath, collected on charged slides, and dried at 37°C overnight.

#### Pimonidazole adduct staining

Tissue sections were obtained from formalin-fixed, paraffin-embedded tumor material and scanned using fluorescent microscopy on a Panoramic 250 Flash system (3DHistech Ltd.) to determine pimonidazole binding. Data were analyzed using Definiens Developer 2.7 Tissue Studio software (Definiens). Pathology hypoxic fraction was represented by the percentage of stained area in viable tumor.

#### Hematoxylin and eosin

Tissue sections were stained with hematoxylin and eosin (H&E) and whole field images were examined using Definiens Developer XD version 2.5 and the Tissue Studio Portal version 4.2 (Definiens AG). Tumors were segmented into viable and necrotic tumor using a threshold technique and the section area percentage necrosis was calculated. The operator was blinded to the OE-MRI data. Next, adjacent sections were scanned using fluorescent microscopy on a Panoramic 250 Flash system (3DHistech Ltd.) to determine pimonidazole binding. Data were analyzed using Definiens Developer 2.7 Tissue Studio software (Definiens). Pathology necrotic fraction was represented by the percentage of stained area in viable tumor, as described elsewhere (33).

### Statistical analysis

GraphPad Prism 7.0 (GraphPad Software; RRID:SCR_002798) was used for all *in vitro* data analyses, with one-way or two-way ANOVA was applied wherever appropriate. IBM SPSS (RRID:SCR_002865) Statistics v.22 was used for all *in vivo* and *ex vivo* data analyses. In all cases, *P*-values of <0.05 were considered significant. Nonparametric statistics were used to compare differences in tumor size, imaging (PET and MRI), and pathology measurements, with correction applied wherever necessary for multiple comparisons.

### Data availability

The data generated in this study are available upon request from the corresponding author.

## Results

### Banoxantrone and atovaquone target hypoxia via distinct mechanisms and have differential cytotoxicity and oxygen consumption profiles *in vitro*

**Figure 1:**
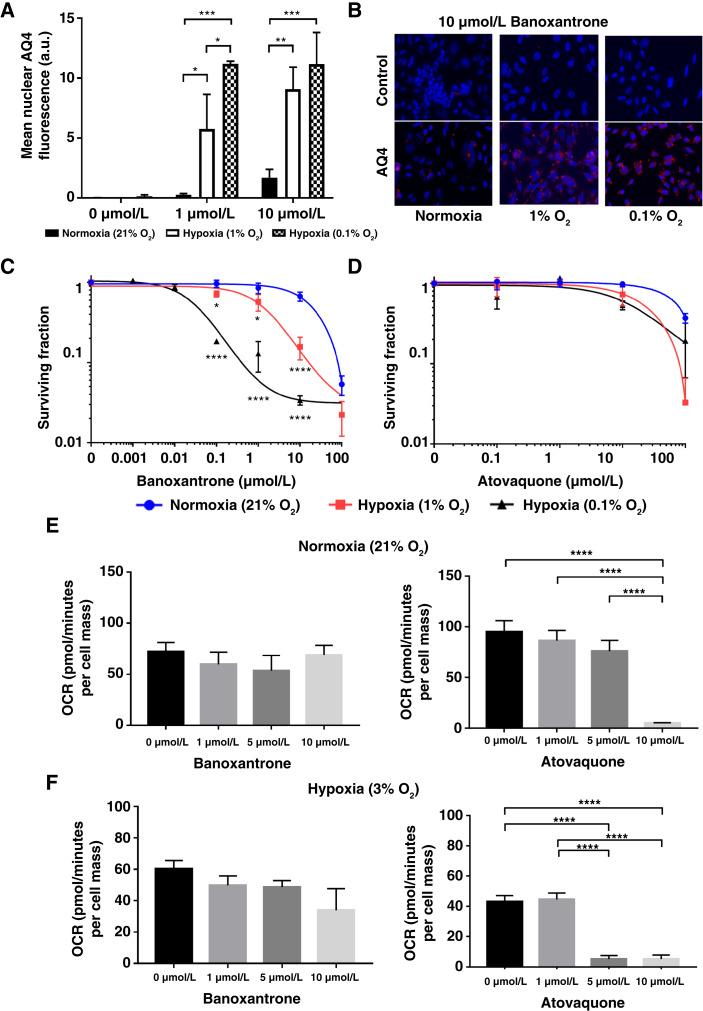
Determining drug mechanism of action in Calu6 cells *in vitro*. **A,** Increased retention of the fluorescent cytotoxic metabolite of banoxantrone, AQ4, as oxygen concentration decreased, with (**B**) example images shown for cells exposed to 10 µmol/L of banoxantrone differing oxygenation conditions. **C** and **D,** Clonogenic survival assays showed that banoxantrone became increasingly cytotoxic with reduced oxygenation whereas the impact of atovaquone on survival was not affected by oxygen level. **E,** A total of 10 µmol/L atovaquone completely ablated OCR under normoxia while banoxantrone had no effect. **F,** Under hypoxia (3% O_2_), OCR ablation was observed at 1 µmol/L atovaquone and while a trend toward reduced OCR was observed for banoxantrone this was not significant.

The Calu6 cells were exposed to banoxantrone or atovaquone *in vitro* under normoxic or increasingly hypoxic (1% and 0.1% oxygen) conditions. Following exposure to banoxantrone, fluorescence associated with the retention of the cytotoxic metabolite of banoxantrone (AQ4) increased consistently with decreasing oxygen but was negligible in normoxic cells (Fig. 1A–C). Banoxantrone caused differential cytotoxicity at concentrations of 10 µmol/L and below dependent on oxygenation profile, becoming increasingly cytotoxic with reduction in oxygen (IC_50_ normoxia, 29 µmol/L; 1% oxygen, 1.5 µmol/L; and 0.1% oxygen, 0.003 µmol/L). In distinction, oxygenation had no significant effect on the response to atovaquone (Fig. 1D).

Mitochondria-specific OCR was assessed on Calu-6 cells treated with or without banoxantrone and atovaquone for 24 hours under normoxia (Fig. 1E) and 3% hypoxia (Fig. 1F). In normoxia (21% O_2_), banoxantrone had no effect on Calu6 OCR but had a trend for dose-dependent OCR reduction in hypoxia (3% O_2_). In distinction, atovaquone completely ablated OCR at concentrations of 1 µmol/L in hypoxia (3% O_2_) and 10 µmol/L in normoxia (21% O_2_). Both drugs elicited effects at doses that caused no change in cell number or cell viability using the SRB protein–binding quantification as a proxy for cell mass (Supplementary Fig. S4).

### OE-MRI detects hypoxia modification induced by the hypoxia-activated prodrug banoxantrone

**Figure 2:**
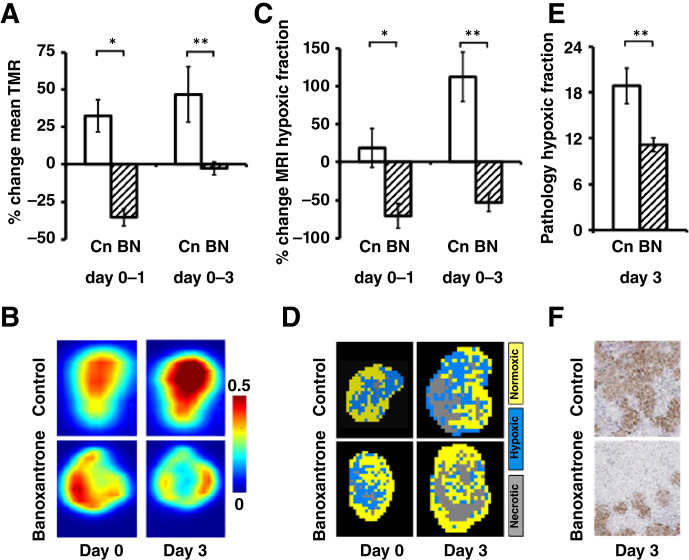
Banoxantrone (BN) induces modification of tumor hypoxia in Calu6 xenografts. **A,** TMR of [^18^F] FAZA uptake is increased from baseline at day 1 and 3 in control tumors (Cn; white bars) but was reduced in drug-treated tumors. **B,** Example, [^18^F] FAZA PET images with greatest hypoxia represented by orange and red. **C,** Tumor MRI hypoxic fraction showed similar relative reduction at day 1 and 3, relative to control tumors. **D,** Example segmented MRI images showing hypoxic (blue), normoxic (yellow), and necrotic (gray) tumor. **E,** Lower percentage of pimonidazole staining was detected in drug-treated tumors at day 3, compared to controls. **F,** Example pimonidazole adduct formation images (×40 magnification).

Previous studies have shown that hypoxia PET tracers can detect the cytotoxic effects of the hypoxia-activated prodrugs TH-302 and SN30000 in combination with radiotherapy *in vivo* (16, 34). We confirmed the differential temporal evolution of banoxantrone-induced changes in mice bearing Calu6 xenografts using [^18^F]FAZA PET imaging at days 0, 1, and 3 following either saline (vehicle; *n* = 4) or banoxantrone treatment (60 mg/kg; *n* = 10). Progressive increase in [^18^F] FAZA uptake, quantified by TMR, was seen in vehicle control tumors over the 3 days. In distinction, banoxantrone treatment caused significant reduction in TMR at day 1, relative to baseline (vehicle 32.4% increase ± 10.8%; treated 35.2% decrease ± 5.7%; *P* < 0.001) and at day 3 (vehicle 46.8% increase ± 18.6%; treated 2.9% decrease ± 4.3%; *P* = 0.009), consistent with targeting of hypoxic tumor cells (Fig. 2A and B).

We then evaluated if OE-MRI could detect and quantify the hypoxia modification induced by banoxantrone. A separate cohort of mice bearing Calu6 xenografts were imaged with MRI, again at days 0, 1, and 3 following either saline (vehicle; *n* = 7) or banoxantrone treatment (60 mg/kg; *n* = 12; Supplementary Fig. S1A).

Consistent with [^18^F]FAZA PET TMR data, progressive increase in MRI hypoxic fraction was observed in vehicle control tumors by day 3. In distinction, banoxantrone treatment caused significant reduction in MRI hypoxic fraction at day 1, relative to baseline (vehicle 18.4% increase ±25.6%; treated 70.3% decrease ± 16.4%; *P* = 0.028) and at day 3 (vehicle 112.5% increase ± 32.7%; treated 52.5 decrease ± 12.0%; *P* = 0.008; Fig. 2C and D), consistent with drug-induced hypoxia modification.

*Ex vivo* validation of these findings was provided using tissue pathology assessment of hypoxic fraction from the mice undergoing OE-MRI. Pimonidazole adduct–formation data were collected at day 3. This confirmed that banoxantrone reduced hypoxia in Calu6 xenograft tumors (vehicle 18.9% ± 2.3%; treated 11.1% ± 0.9%; *P* = 0.009; Fig. 2E and F). Collectively, these data validate the ability of combined OE-MRI and DCE-MRI to detect and quantify hypoxia-activated cytotoxic therapy effects.

### OE-MRI detects hypoxia-modification induced by the oxygen consumption–modifier atovaquone

**Figure 3:**
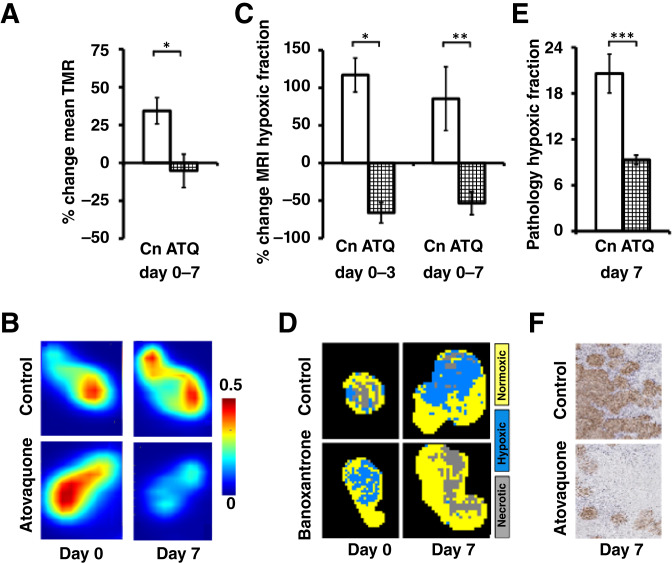
Atovaquone (ATQ) induces modification of tumor hypoxia in Calu6 xenografts. **A,** TMR [^18^F] FAZA uptake is increased from baseline at day 7 in control tumors (Cn; white bars) but was reduced in drug-treated tumors. **B,** Example [^18^F] FAZA PET images, with greatest hypoxia represented by orange and red. **C,** Tumor MRI hypoxic fraction showed similar relative reduction at day 3 and 7, relative to control tumors. **D,** Example segmented MRI images showing hypoxic (blue), normoxic (yellow), and necrotic (gray) tumor. **E,** Lower percentage of pimonidazole staining was detected in drug-treated tumors at day 7, compared to controls. **F,** Example pimonidazole adduct formation images (×40 magnification).

Previous immunofluorescence data have shown that atovaquone reduces hypoxia *in vitro* in spheroids derived from multiple cell lines and in xenograft models, including NSCLC tumors, within 7 days of treatment that leads to improved radiosensitivity (25). We confirmed that hypoxia modification by atovaquone could also be detected *in vivo* within 7 days in mice bearing Calu6 xenografts using [^18^F]FAZA PET at days 0 and 7 following vehicle (*n* = 8) or atovaquone (treated; *n* = 11). TMR was reduced in atovaquone-treated tumors at day 7, relative to baseline (vehicle 34.4% increase ± 6.6%; treated 5.2% decrease ± 11.1%; *P* = 0.017), consistent with increasing oxygen availability (Fig. 3A and B).

We then evaluated if OE-MRI could detect and quantify atovaquone-induced changes. Studies followed the methods described for banoxantrone, except that MRI of vehicle (*n* = 9) and atovaquone-treated (*n* = 12) Calu6 tumor–bearing mice was undertaken at days 0, 3, and 7 in line with previous studies (25). Again, a separate cohort of mice was used for MRI and PET (Supplementary Fig. S1B). MRI hypoxic fraction was reduced in atovaquone-treated tumors at day 3, relative to baseline (vehicle 115.4% increase ± 23.6%; treated 68.4% decrease ± 12.5%; *P* = 0.001) and at day 7 (vehicle 85.5% increase ± 42.3%; treated 53.4% decrease ± 15.3%; *P* = 0.002; Fig. 3C and D).

Pimonidazole adduct formation in tumor samples collected at day 7 confirmed that atovaquone reduced hypoxia in treated xenograft tumors (vehicle 20.6% ± 2.5%; treated 9.3% ± 0.6%; *P* < 0.001; Fig. 3E and F). Collectively, these data validate the ability of combined OE-MRI and DCE-MRI to detect and quantify hypoxia modification resulting from altering OCR.

### OE-MRI detects hypoxia modification in tumor models with differing levels of baseline hypoxia

We tested if OE-MRI detection of drug-induced hypoxia modification was consistent across different tumor models, using a tumor model with different growth characteristics and lower baseline levels of hypoxia than Calu6. U87 glioblastoma xenografts were chosen from a screen of four xenograft models (HCT116; KHT; MDA-MB-231, U87; Supplementary Fig. S1C), as the U87 xenografts had the lowest hypoxic fraction on MRI at a size range of around 200 mm^3^. Untreated U87 tumors had a shorter volume doubling time (*T*_*D*_) of 3.3 days (*n* = 14; starting size range 180–480 mm^3^) than Calu6 tumors, which had a *T*_*D*_ of 7.8 days (*n* = 22; starting size range 198–472 mm^3^; *P* < 0.001; Supplementary Fig. S5).

**Figure 4:**
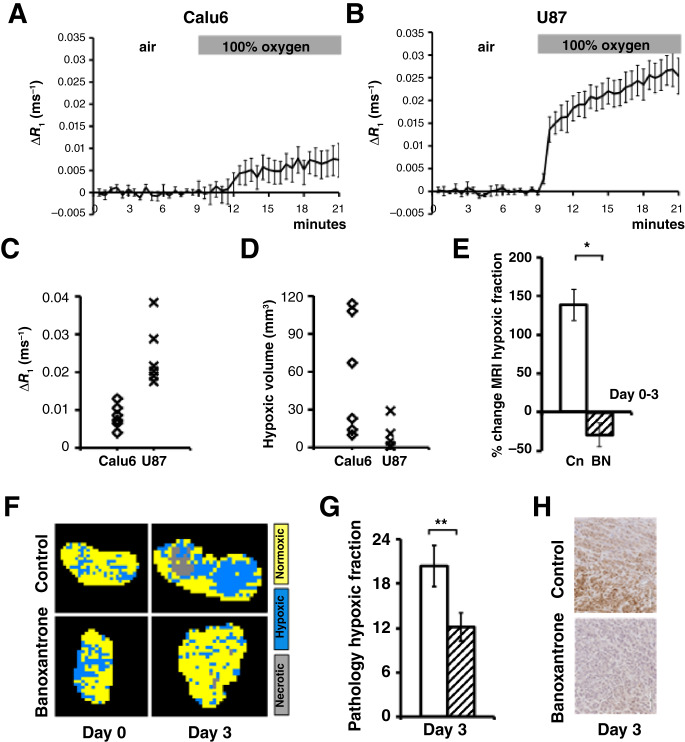
Banoxantrone (BN) hypoxia modification is replicated in U87, a xenograft model with different growth and hypoxia characteristics. U87 xenografts were less hypoxic than Calu6 xenografts. The group average ΔR_1_ for size matched (**A**) Calu6 was less than that for (**B**) U87 xenografts. Error bars are SEM. Significant difference between (**C**) group ΔR_1_ and (**D**) group MRI hypoxic volume were observed between the two tumor models. Examining the U87 xenografts further, (**E**) U87 Cohort reduction in MRI hypoxic fraction was seen at day 3 in treated drug tumors, relative to control tumors (Cn; white bars), with (**F**) U87 example segmented MRI images showing hypoxic (blue), normoxic (yellow), and necrotic (gray) tumor. **G,** Lower percentage of pimonidazole staining was detected in drug-treated U87 tumors at day 3, compared to controls. **H,** Example pimonidazole adduct formation images (×40 magnification).

We confirmed that OE-MRI detected differing levels of tumor oxygenation and hypoxia in U87 and Calu6 xenograft models. Group average whole tumor Δ*R*_1_ are shown for size matched Calu6 (*n* = 6; Fig. 4A) and U87 (*n* = 6; Fig. 4B) xenografts. Calu6 xenografts had mean Δ*R*_1_ of 0.0087 seconds^−1^ (SD 0.0015 seconds^−1^) and an MRI hypoxic volume of 56 mm^3^ (SD 23.5 mm^3^), whereas U87 xenografts had mean Δ*R*_1_ of 0.0244 seconds^−1^ (SD 0.0032 seconds^−1^) and an MRI hypoxic volume of 8 mm^3^ (SD 4.5 mm^3^). The U87 tumor Δ*R*_1_ were significantly greater than Calu6 (unpaired *t* test *P* = 0.003; Fig. 4C) and U87 hypoxic volumes were significantly lower than Calu6 (unpaired *t* test *P* = 0.036; Fig. 4D).

OE-MRI detected increase in MRI hypoxic fraction in U87 untreated tumors (*n* = 9) at day 3. In distinction, banoxantrone treatment (*n* = 7) caused significant relative reduction in MRI hypoxic fraction at day 3 (vehicle 138.5% increase ± 42.6%; treated 29.0 decrease ± 15.8%; *P* = 0.004; Fig. 4E and F; Supplementary Fig. S1D), consistent with the drug-induced hypoxia modification. *Ex vivo* validation of these findings was provided using tissue pathology assessment of hypoxic fraction in the same mice. This confirmed that banoxantrone reduced hypoxia in the treated U87 xenograft tumors (vehicle 20.4% ± 2.8%; treated 12.2% ± 1.9%; *P* = 0.046; Fig. 4G and H). Collectively, these data show that OE-MRI can detect and quantify therapy-induced changes in hypoxia consistently in xenograft models with different growth rates and different levels of pretreatment hypoxia.

### Combined OE-MRI and DCE-MRI differentiates drug mechanism of action for banoxantrone and atovaquone

**Figure 5:**
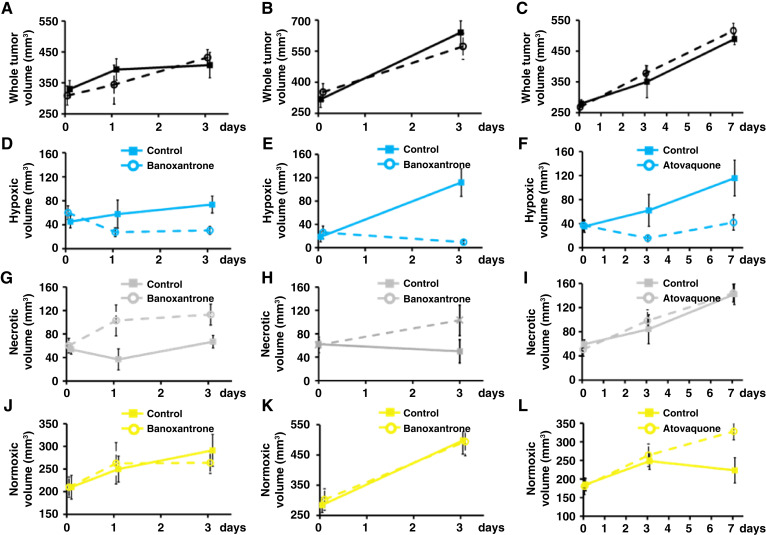
MRI elucidates differential drug mechanism of action for banoxantrone and atovaquone. Banoxantrone did not cause growth inhibition in (**A)** Calu6 xenografts or (**B**) U87 xenografts. Atovaquone did not cause growth inhibition in (**C)** Calu6 xenografts. **D** and **E,** Calu6 and U87 xenografts show reduction in volume of hypoxic tumor with banoxantrone by day 3. Similarly, (**F**) Calu6 xenografts show reduction in volume of hypoxic tumor with atovaquone by day 7. **G** and **H,** Calu6 and U87 xenografts show companion increases in volume of necrotic tumor, whereas (**I**) no change in volume of necrotic tumor is detected in Calu6 xenografts treated with atovaquone. **J** and **K,** No change in volume of normoxic tumor is detected in Calu6 or U87 xenografts treated with banoxantrone, whereas (**L**) Calu6 xenografts treated with atovaquone show increase in normoxic tumor. Changes described are all relative to baseline.

Anatomical imaging enables dynamic tracking of changes in tumor volume. No growth inhibition was detected between vehicle control and treated xenografts with either banoxantrone in Calu6 and U87 or with atovaquone in Calu6, using MRI measurement of whole tumor volume (Fig. 5A–C). Performing multimodal functional MRI enables mapping of tissue subregions with distinct tumor microenvironments or “imaging habitats” (35, 36). We hypothesized that combined OE-MRI and DCE-MRI imaging would not only identify and quantify the hypoxia modification induced by each drug but would also differentiate differences in drug mechanism of action by evaluating habitats.

The MRI hypoxic volume (voxels that enhance with DCE-MRI but are refractory to oxygen challenge with OE-MRI) was reduced with banoxantrone in both Calu6 (*P* = 0.013 at day 3; Fig. 5D) and U87 xenografts (*P* = 0.019 at day 3; Fig. 5E), relative to baseline. Similarly, the volume of hypoxic tumor was reduced with atovaquone in Calu6 xenografts, relative to baseline (*P* = 0.022 at day 7; Fig. 5F). Unsurprisingly, these changes in MRI hypoxic volume mirrored changes in MRI hypoxic fraction.

However, additional insight into drug mechanism was provided by tracking changes in necrotic and normoxic volumes. Tumors treated with banoxantrone had increase in the necrotic volume, relative to baseline for Calu6 (*P* = 0.039; Fig. 5G) and U87 xenografts (*P* = 0.048; Fig. 5H) at day 3, but no change in normoxic volume, relative to baseline (Fig. 5J and K). These data were consistent with hypoxic tissue being converted into necrotic tissue due to prodrug activation and resultant cytotoxic action. *Ex vivo* validation was provided through H&E pathology assessment of necrotic fraction in both Calu6 (Supplementary Fig. S6A) and U87 tumors (Supplementary Fig. S6B).

In distinction, atovaquone did not alter the necrotic volume (Fig. 5I) but induced significant increase in the normoxic volume of Calu6 xenografts, at day 7 relative to baseline (*P* = 0.024; Fig. 5L), consistent with hypoxic tissue being converted into normoxic tissue due to improved oxygen availability following reduction in oxygen consumption rate. These data show that combined OE-MRI and DCE-MRI can track transformation of tumor subregions from one habitat to another, dependent on the drug mechanism of action. Specifically, MRI showed *in vivo* tracking of banoxantrone conversion of hypoxic tumor habitats into necrotic tumor and atovaquone conversion of hypoxic tumor habitats into normoxic tumor.

## Discussion

Several therapeutic agents can modify tumor hypoxia to provide clinical benefit (1). However, drug development and clinical trial evaluation have been hampered by the lack of available, affordable, and validated clinical tools that identify hypoxia pretreatment and quantify the change in hypoxia on therapy (10).

Imaging offers a solution to this unmet need. OE-MRI measures the oxygen-induced change in tissue proton *R*_1_ induced when subjects switch from breathing air to inhaling 100% oxygen or carbogen (18). Increase in *R*_1_ has been detected in well-perfused well-oxygenated organ tissues in numerous studies (37, 38). Multiple investigators have shown that oxygen inhalation increases *R*_1_ in viable tumor tissue (39–45), whereas there is negligible signal change observed in hypoxic subregions. Our previous work used pimonidazole assays to validate that perfused tumor regions refractory to oxygen enhancement identify tissue hypoxia and can quantify its extent (20, 21, 23). This multimodal MRI approach—combining OE-MRI and DCE-MRI—has been shown feasible in clinical studies of patients with cancer (23, 46, 47) and able to monitor hypoxia modification induced by (chemo)-radiotherapy in Calu6 and U87 xenograft tumors and in patients with NSCLC (23).

Here, we report the first application of OE-MRI to assess drugs designed specifically to modify tumor hypoxia. We evaluated the effects of the prodrug banoxantrone, which inhibits topoisomerase II to cause direct cytotoxic effects in hypoxic cells, alongside atovaquone, which inhibits mitochondrial complex III of the electron transport chain to decrease the rate of oxygen consumption in tumors. MRI data were acquired in two animal models, Calu6 and U87, chosen following a screen of multiple xenograft models as these represented models with differing levels of pretreatment hypoxia. Our data revealed three main findings that support the clinical translation (48) of OE-MRI biomarkers.

First, we demonstrated that combined OE-MRI and DCE-MRI could detect hypoxia modification in studies of two hypoxia-modifying agents. Experiments in Calu6 xenografts confirmed that OE-MRI hypoxic fraction was reduced with both banoxantrone and atovaquone. Data were validated using another imaging technology ([^18^F]FAZA-PET performed on different animals, allowing cohort level comparison) and the gold standard *ex vivo* pathology technology (hypoxic fraction assessed by pimonidazole adduct formation in the mice undergoing MRI). We showed for the first time that changes in MRI hypoxic fraction were significant and occurred in the same timeframe as changes detected by PET imaging and pathology. Findings were confirmed in a second model (U87) with different growth characteristics and lower levels of pretreatment hypoxia.

Second, we highlighted the limitations of using tumor size to monitor targeted therapies. Clinical trials of hypoxia-modifying agents use RECIST and other size-based approaches to monitor biological responses from hypoxia-modifying therapies. Here, we show that MRI evaluation of tumor volumes failed to detect response to therapy for either hypoxia-modifying drug in either tumor model during a period where drugs exhibited mechanistic activity.

Third, we demonstrated that combined OE-MRI and DCE-MRI could distinguish the different mechanisms of action of banoxantrone and atovaquone. Tumors have spatially distinct tissue subregions that include hypoxic tissue, well-perfused normoxic tissue and non-perfused necrotic tissue. Multiparametric MRI can be used to track changes in these subregions in an approach known as habitat imaging (49). Combined OE-MRI and DCE-MRI demonstrated that banoxantrone-treated tumors exhibited hypoxia modification along with increase in necrotic volume, relative to control. This is consistent with the banoxantrone active drug AQ4 having a cytotoxic effect in hypoxic tumor subregions (30, 31). In distinction, atovaquone-treated tumors exhibited hypoxia modification along with increase in normoxic volume, relative to control. This is consistent with conversion of hypoxic tissue into well-oxygenated tissue following modification of tissue oxygen consumption. These data show that multimodal MRI provided additional mechanistic information over that obtained by hypoxia PET imaging alone.

Study limitations include the fact that the two validation techniques (PET and immunohistochemistry) may measure different levels of hypoxia to that measured by OE-MRI. This needs further study elucidation. Further, perfusion will affect the uptake of PET tracers and hence the variable nature of necrosis in each tumor can also have an effect on how quantification of hypoxia based on [^18^F]FAZA-PET imaging (50). As this was a first proof-of-principle that OE-MRI could inform on mechanism and antitumor effects of hypoxia modifying agents we used simple preclinical xenograft models. Findings reported here require replication in other models such as orthotopic and patient-derived models. Finally, we choose to examine two therapies that are well understood in the relevant clinical models used here and have different mechanisms of hypoxia modification, rather than agents that are about to enter later clinical trial evaluation.

Data reported here support translation of OE-MRI into the clinic. There has been renewed interest in detecting hypoxia modification following a recent trial in HPV positive oropharyngeal cancer where PET data helped identify benefits of radiotherapy dose de-escalation based on absence of hypoxia in patients with oropharyngeal cancers (51). Work from our group has since shown that OE-MRI combined with DCE-MRI can identify hypoxia modification in the same clinical setting (52) and identify differential biological response patterns in primary tumors and nodal metastases for each patient. Similar approaches to those already demonstrating benefit with assessment of radiation are envisaged in trials of hypoxia-modifying agents.

In conclusion, OE-MRI should now be evaluated in early phase clinical trials to confirm the presence of hypoxia in patients’ tumors and to map and quantify biological response induced by therapies. Further work in adequately powered patient cohorts will determine if these combined OE-MRI and DCE-MRI biomarkers have a predictive use and can also act to select patients for later phase clinical trials.

## Supplementary Material

Supplementary Figure S1 Supplementary Figure S1 shows a summary of the cohorts of xenografts used in each experiment.

Supplementary Figure S2 Supplementary Figure S2: Confirmation that oxygen increases tumor R1.

Supplementary Figure S3 Supplementary Figure S3 shows the MRI analysis steps.

Supplementary Figure S4 Supplementary Figure S4 confirms that there was no drug effect on cell number or viability.

Supplementary Figure S5 Supplementary Figure S5 compares the natural history growth of Calu6 and U87 xenografts.

Supplementary Figure S6 Supplementary Figure S6 shows that banoxantrone induces tumor necrosis.
