# Supplementary material for: Combined Oxygen-Enhanced MRI and Perfusion Imaging Detect Hypoxia Modification from Banoxantrone and Atovaquone and Track Their Differential Mechanisms of Action
Source: Cancer Res Commun. 2024 Oct 1;4(10):2565–74. doi: 10.1158/2767-9764.CRC-24-0315 (PMC11443776; doi:10.1158/2767-9764.CRC-24-0315)

Supplementary Figure S1: Summary of the cohorts of xenografts used in each experiment. Mouse numbers are shown along with the imaging modalities used in each experiment.

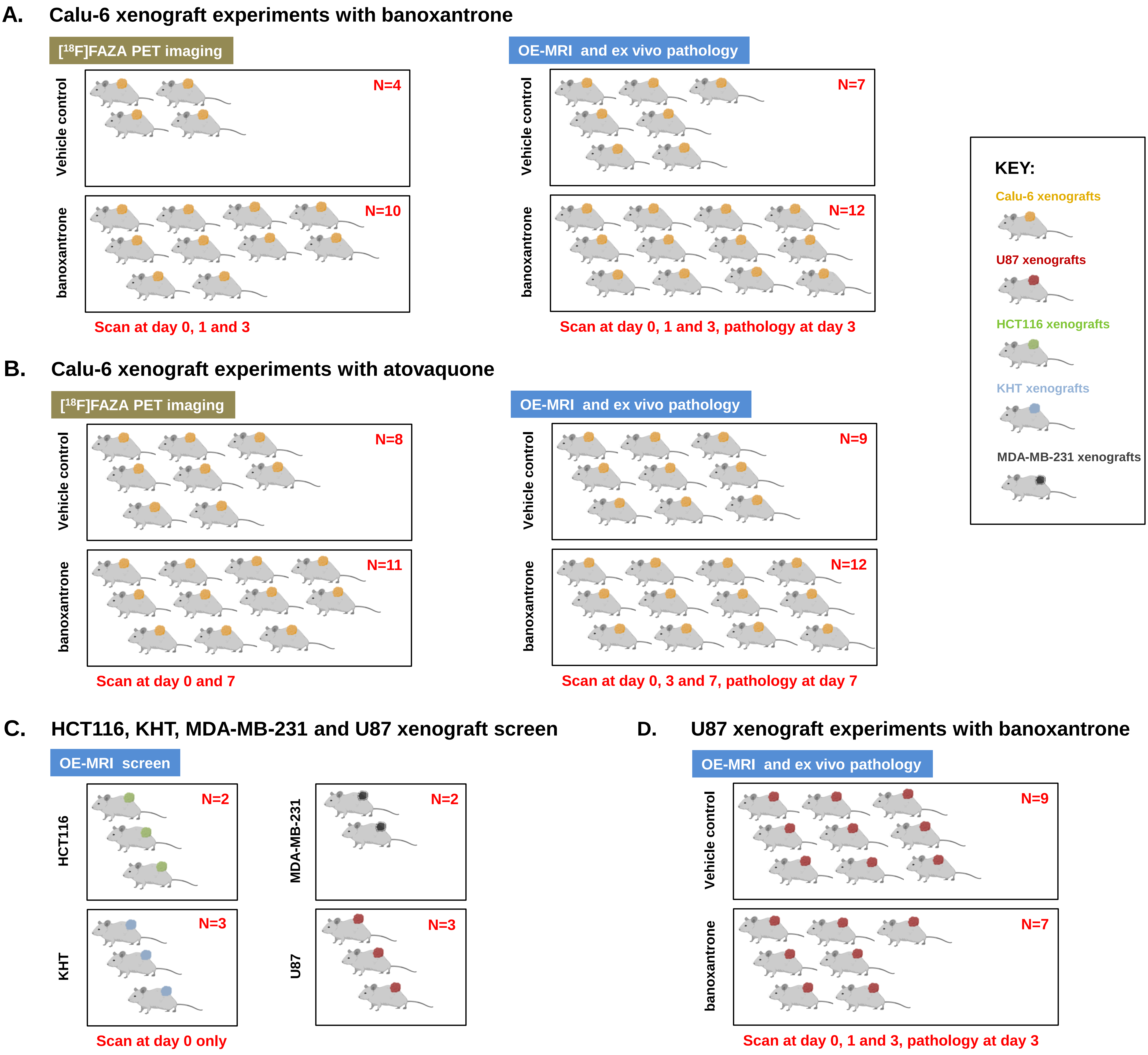

Supplement: Supplementary Figure S1 — shows a summary of the cohorts of xenografts used in each experiment. [file crc-24-0315_supplementary_figure_s1_suppsf1.pdf]
