# Supplementary material for: Combined Oxygen-Enhanced MRI and Perfusion Imaging Detect Hypoxia Modification from Banoxantrone and Atovaquone and Track Their Differential Mechanisms of Action
Source: Cancer Res Commun. 2024 Oct 1;4(10):2565–74. doi: 10.1158/2767-9764.CRC-24-0315 (PMC11443776; doi:10.1158/2767-9764.CRC-24-0315)

Supplementary Figure S2: Confirmation that oxygen increases tumor R_1.
A) In the absence of a gas challenge, there is no measurable enhancement, measured by change in longitudinal relaxation rate (ΔR_1). B) In distinction, following gas challenge with 100% oxygen there is clear evidence of tumor enhancement in both Calu6 and U87 xenograft models.

A. No oxygen-enhancement detected in the tumor when breathing air only

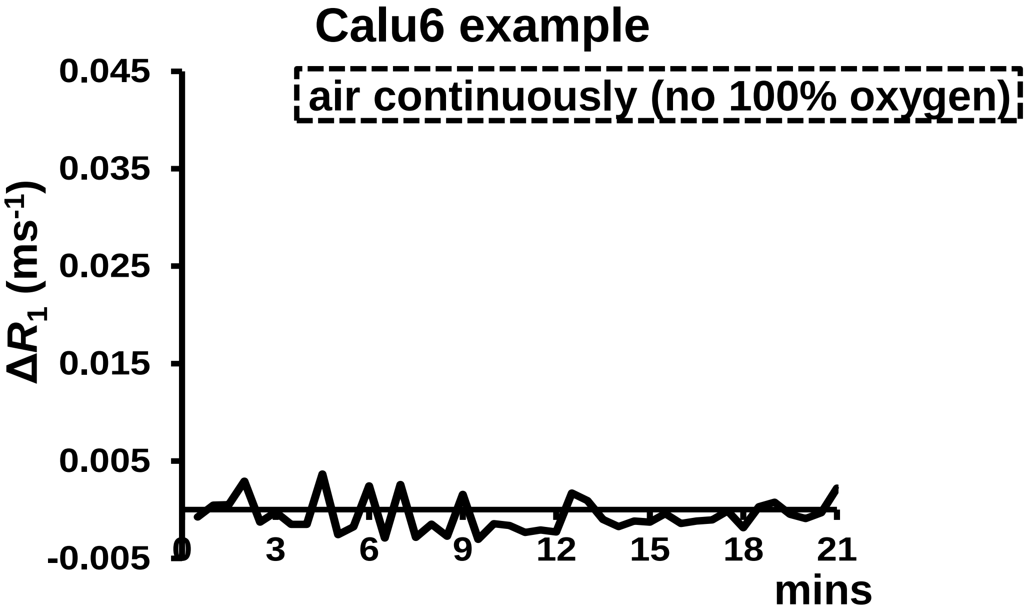

B. Positive median oxygen-enhancement detected in the tumor

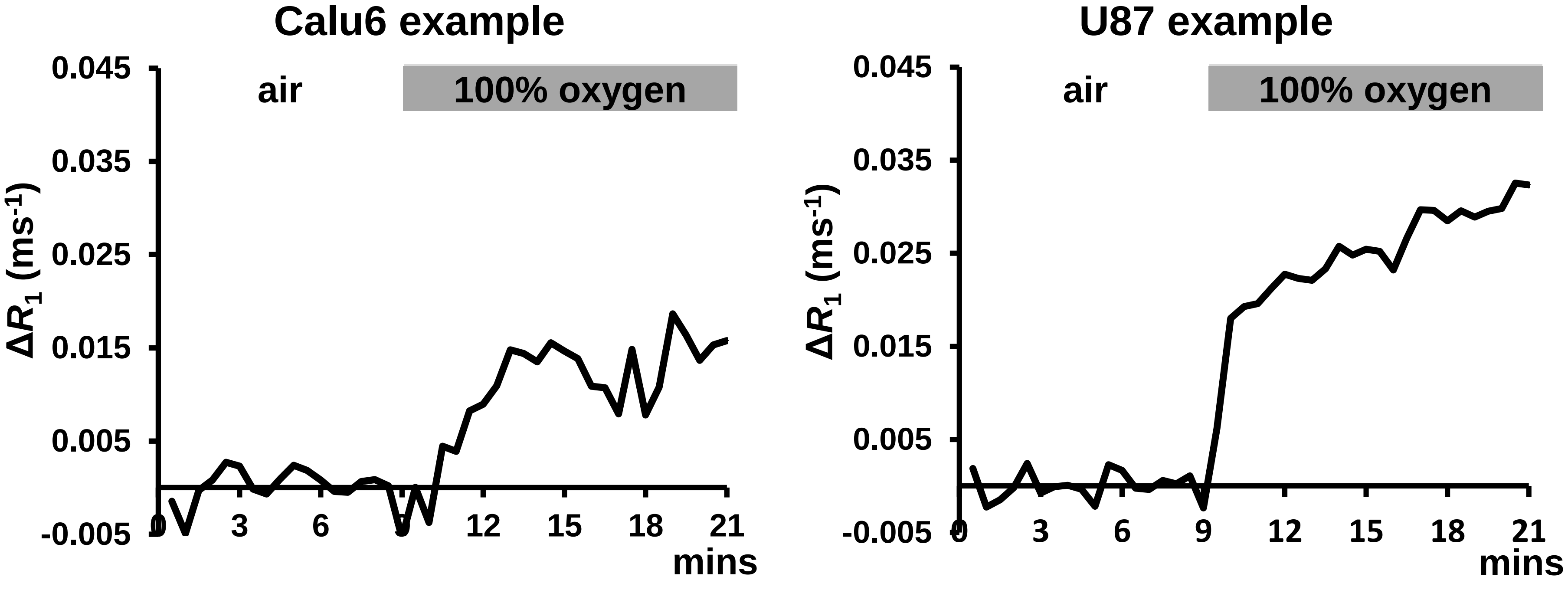

Supplement: Supplementary Figure S2 — Confirmation that oxygen increases tumor R1. [file crc-24-0315_supplementary_figure_s2_suppsf2.pdf]
