# Supplementary material for: Combined Oxygen-Enhanced MRI and Perfusion Imaging Detect Hypoxia Modification from Banoxantrone and Atovaquone and Track Their Differential Mechanisms of Action
Source: Cancer Res Commun. 2024 Oct 1;4(10):2565–74. doi: 10.1158/2767-9764.CRC-24-0315 (PMC11443776; doi:10.1158/2767-9764.CRC-24-0315)

Supplementary Figure S3: MRI analysis step summary. Quantitative OE-MRI and DCE-MRI tumor ΔR_1 maps were binarized to distinguish perfused Oxy-E (normoxic), perfused Oxy-R (hypoxic) and non-perfused voxels in co-localized images. Example OE-MRI and DCE-MRI acquisitions with corresponding maps are shown for one xenograft tumor. The voxel count was measured for each of the three categories, either on a single slice (when deriving hypoxic fraction) or throughout the whole 3D volume (when deriving normoxic, hypoxic or necrotic volumes).

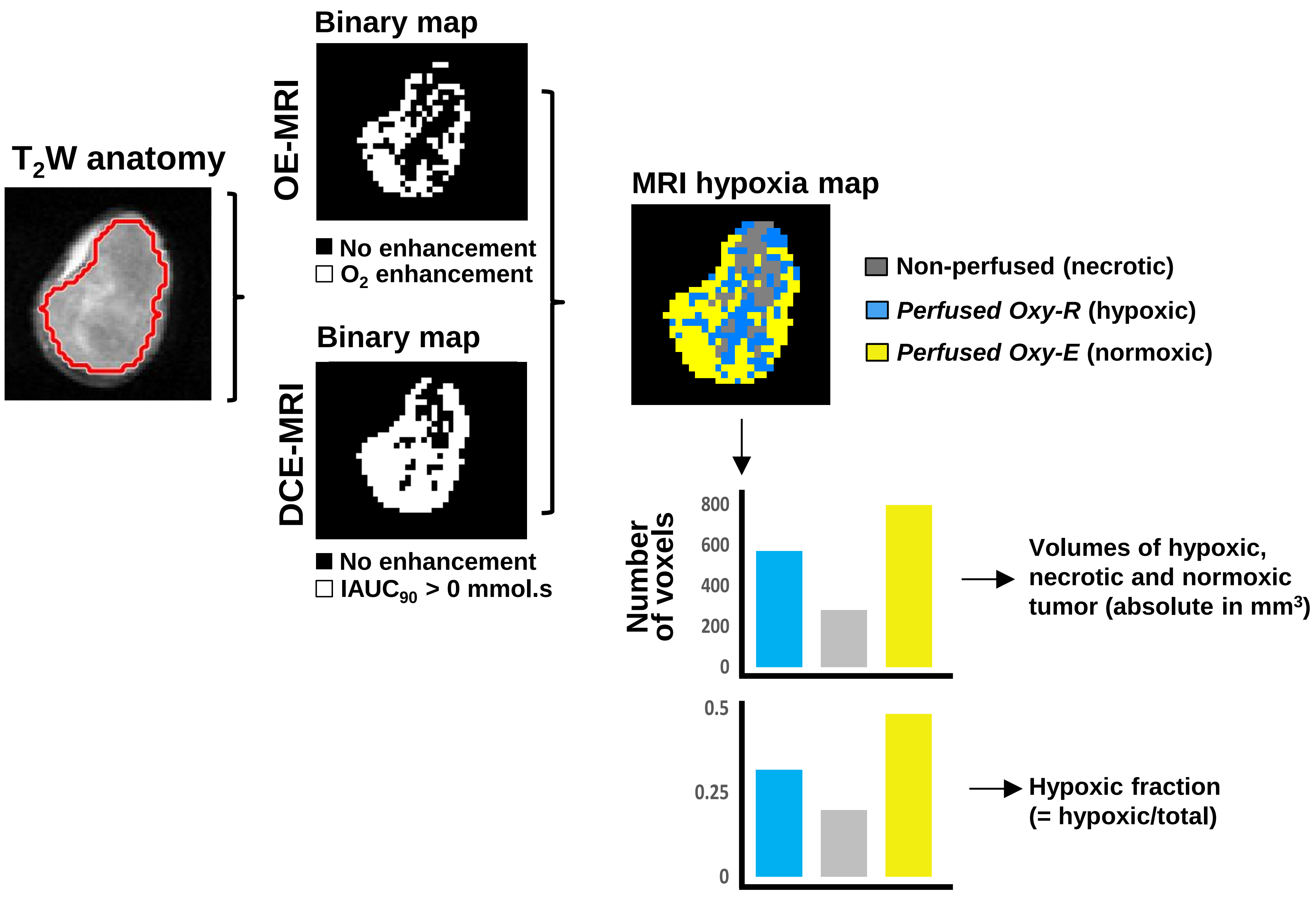

Supplement: Supplementary Figure S3 — shows the MRI analysis steps. [file crc-24-0315_supplementary_figure_s3_suppsf3.pdf]
