# Supplementary material for: Combined Oxygen-Enhanced MRI and Perfusion Imaging Detect Hypoxia Modification from Banoxantrone and Atovaquone and Track Their Differential Mechanisms of Action
Source: Cancer Res Commun. 2024 Oct 1;4(10):2565–74. doi: 10.1158/2767-9764.CRC-24-0315 (PMC11443776; doi:10.1158/2767-9764.CRC-24-0315)

**Supplementary Figure S4: No drug effect on cell number or viability.** Sulphorhodamine B assay results on Calu-6 cells subjected to mitochondrial specific OCR measurements. Banoxantrone and atovaquone had no cytotoxic effects (using a 1-way ANOVA) on Calu-6 cell lines in normoxia (21% $O_2$) or hypoxia (3% $O_2$) for concentrations ranging up to 10 µM.

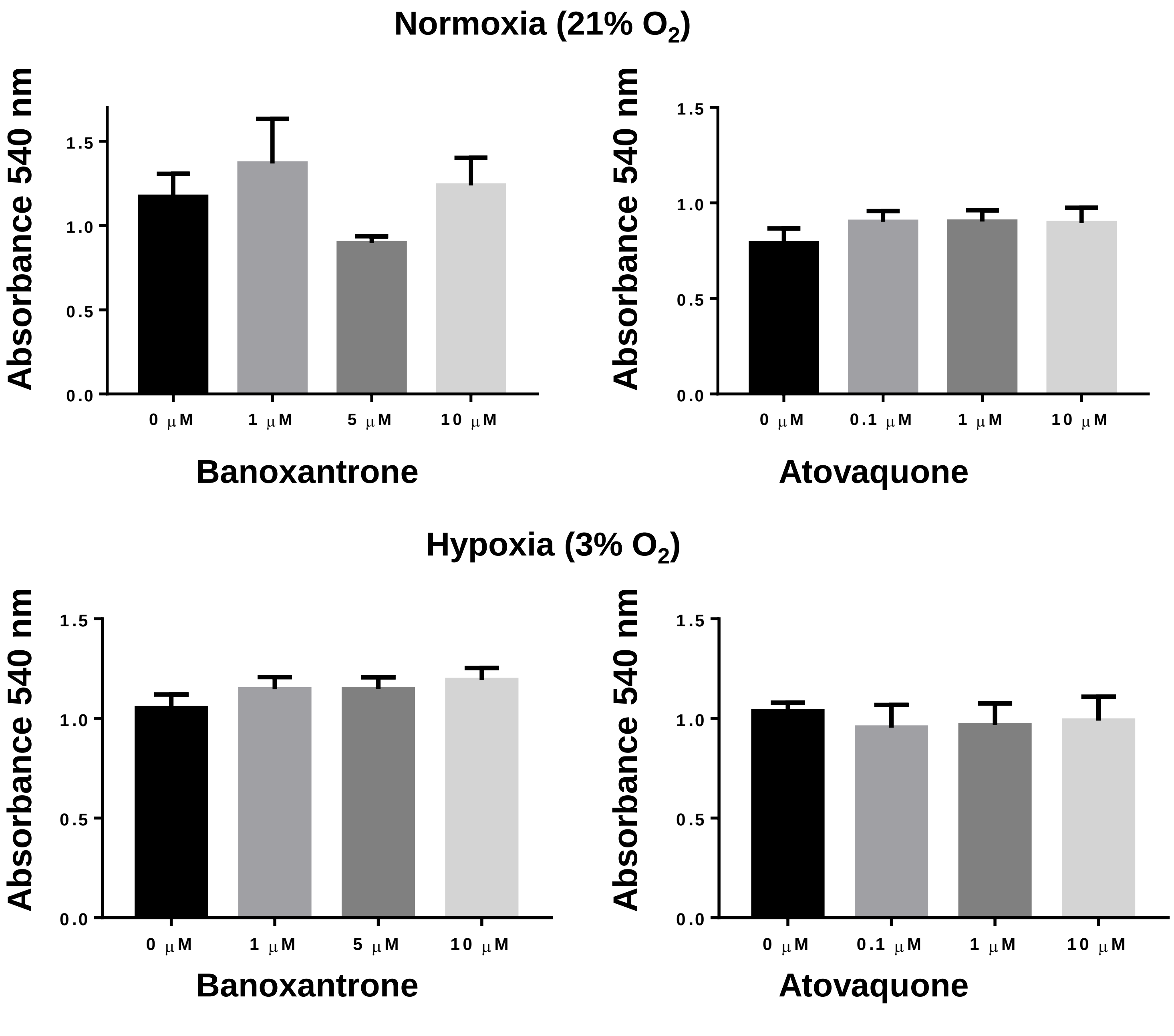

Supplement: Supplementary Figure S4 — confirms that there was no drug effect on cell number or viability. [file crc-24-0315_supplementary_figure_s4_suppsf4.pdf]
