# Supplementary material for: Combined Oxygen-Enhanced MRI and Perfusion Imaging Detect Hypoxia Modification from Banoxantrone and Atovaquone and Track Their Differential Mechanisms of Action
Source: Cancer Res Commun. 2024 Oct 1;4(10):2565–74. doi: 10.1158/2767-9764.CRC-24-0315 (PMC11443776; doi:10.1158/2767-9764.CRC-24-0315)

Supplementary Figure S5: Comparative natural history growth of Calu6 and U87 xenografts. The U87 tumors (N=12; from the screen cohort and the vehicle cohort in Supplementary Figure S1c-d) generally had faster growth rates than Calu6 tumors (N=16 from the two vehicle cohorts in Supplementary Figure S1A-B).

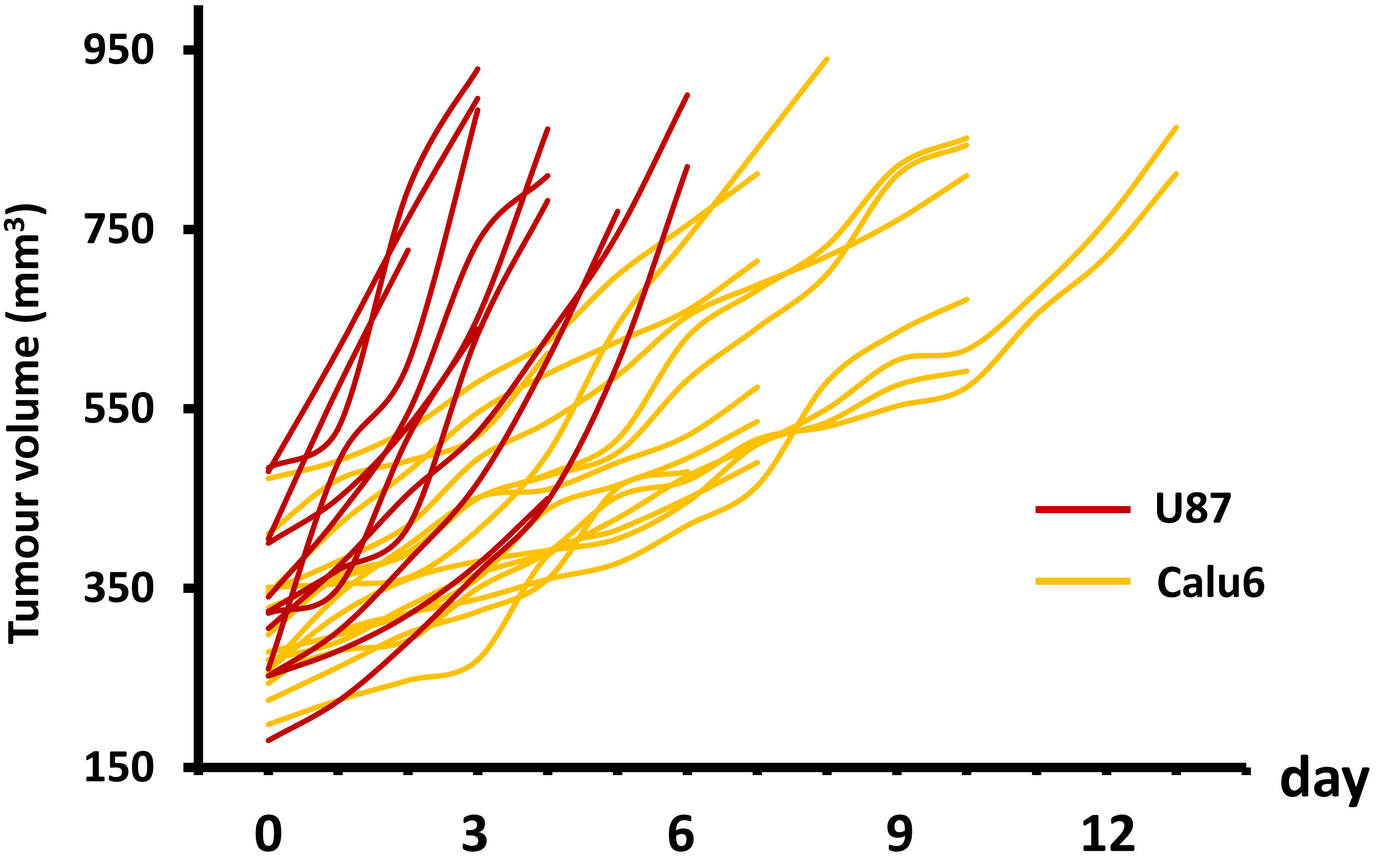

Supplement: Supplementary Figure S5 — compares the natural history growth of Calu6 and U87 xenografts. [file crc-24-0315_supplementary_figure_s5_suppsf5.pdf]
