# Supplementary material for: Combined Oxygen-Enhanced MRI and Perfusion Imaging Detect Hypoxia Modification from Banoxantrone and Atovaquone and Track Their Differential Mechanisms of Action
Source: Cancer Res Commun. 2024 Oct 1;4(10):2565–74. doi: 10.1158/2767-9764.CRC-24-0315 (PMC11443776; doi:10.1158/2767-9764.CRC-24-0315)

Supplementary Figure S6: Banoxantrone induces tumor necrosis. Ex vivo validation that tumor hypoxia modification induced by banoxantrone and detected by combined OE-MRI and DCE-MRI induces tumor necrosis. Higher percentage necrosis was detected in treated tumors at day 3, compared to vehicle in A) Calu6 xenografts with B) sample H&E images (X40 magnification). Comparable data were observed in C) U87xenografts with D) sample H&E images (X40 magnification). Data correspond to the MRI data in Figure 5G-I.

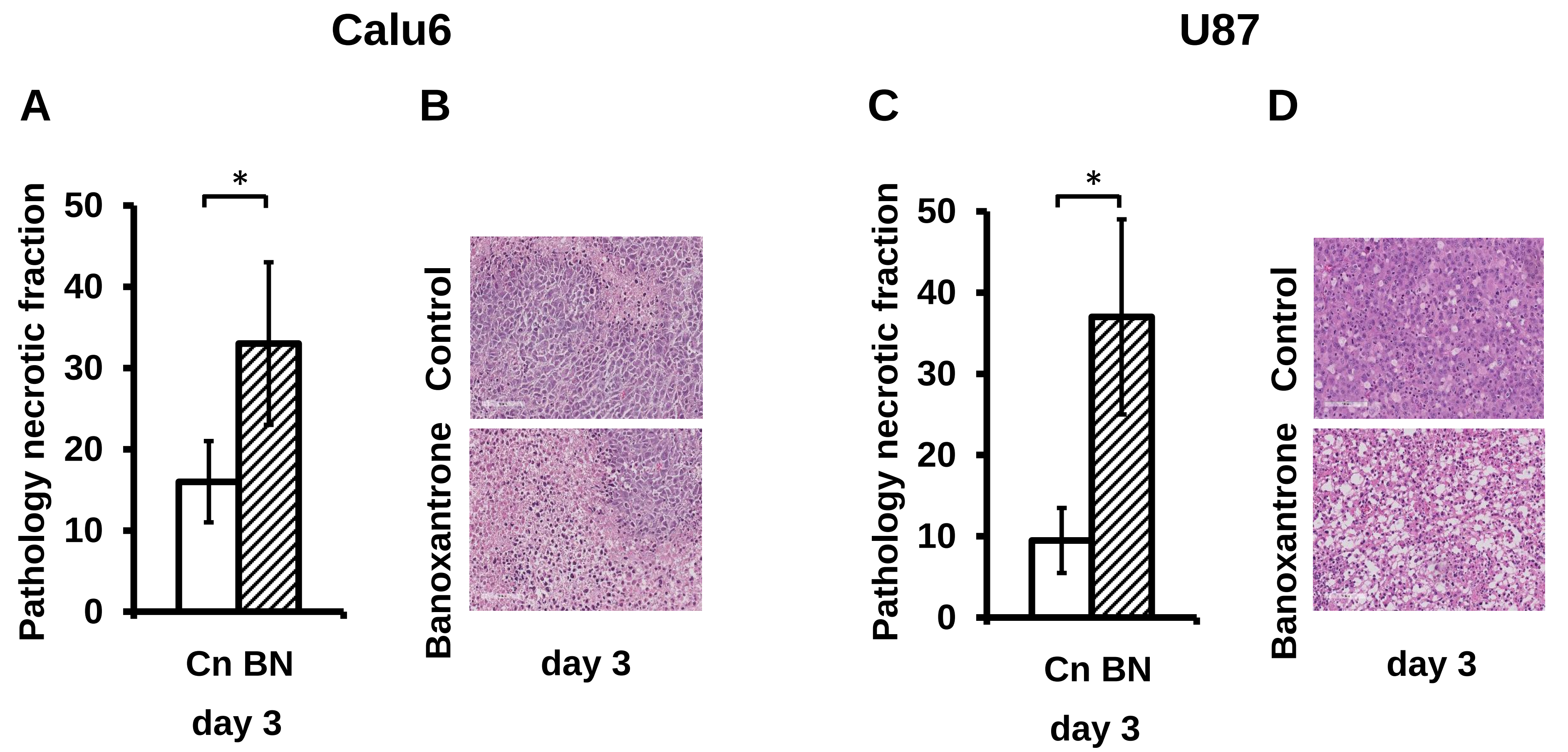

Supplement: Supplementary Figure S6 — shows that banoxantrone induces tumor necrosis. [file crc-24-0315_supplementary_figure_s6_suppsf6.pdf]
